# Classification of Adolescent Drinking via Behavioral, Biological and Environmental Variables: A Machine Learning Approach With Bias Control

**DOI:** 10.1111/adb.70188

**Published:** 2026-09-11

**Authors:** Ruobing Liu, Mohamed Azzam, Nicole L. Zabik, Shibiao Wan, Jennifer Urbano Blackford, Jieqiong Wang

**Affiliations:** ^1^ Department of Neurological Sciences University of Nebraska Medical Center Omaha Nebraska USA; ^2^ Munroe‐Meyer Institute for Genetics and Rehabilitation University of Nebraska Medical Center Omaha Nebraska USA; ^3^ Department of Genetics, Cell Biology and Anatomy University of Nebraska Medical Center Omaha Nebraska USA

**Keywords:** adolescent alcohol use, alcohol drinking classification, brain measures, machine learning

## Abstract

In 2024, approximately 30% of US adolescents reported having consumed alcohol at least once in their lifetime, with about 25% of these individuals engaging in binge drinking. Adolescent alcohol use is associated with neurodevelopmental impairments, elevated risk of later alcohol use and mental health disorders. Therefore, it is important to identify the variables driving adolescent alcohol use and leverage them for early identification and targeted intervention. Previous studies have typically developed machine learning classification models that use neuroimaging data in combination with limited clinical measurements. Neuroimaging data are expensive and difficult to obtain at scale, whereas clinical measures are more practical for large‐scale screening due to their low cost and widespread accessibility. However, clinical‐only approaches for alcohol drinking classification remain largely underexplored. Furthermore, prior studies have often focused on adults, limiting generalizability to the broader adolescent population. Additionally, confounding factors such as age and substance use, which are strongly correlated with alcohol consumption, have often been inadequately addressed, potentially inflating classification performance. Finally, class imbalance remains a persistent challenge, with prior attempts yielding only limited improvements. To address these limitations, we propose *FocalTab*, a framework that integrates TabPFN with focal loss for robust generalization and effective mitigation of class imbalance. The approach also incorporates an initial preprocessing step to remove confounding factors to account for age and substance use. We compare *FocalTab* against state‐of‐the‐art methods across different variable selections and dataset settings. *FocalTab* achieves the highest accuracy (0.841 ± 0.011) and specificity (0.803 ± 0.055) in the most stringent setting, in which both age and substance use variables were excluded, whereas competing models drop to near‐chance specificity (12%–24%). We further applied SHapley Additive exPlanations (SHAP) analysis to identify key clinical predictors of drinker classification, supporting enhanced screening and early intervention.

## Graphical abstract

Machine learning models for adolescent alcohol use screening often rely on costly neuroimaging and are inflated by confounds such as age and substance use. *FocalTab*, which combines TabPFN with focal loss and a confound‐removal step, can classify drinkers from low‐cost clinical measures alone. In the most stringent setting, excluding age and substance use, *FocalTab* attains the highest accuracy (0.841) and specificity (0.803). Meanwhile, competing models fall to near‐chance specificity, and SHAP identifies key clinical predictors for early intervention.

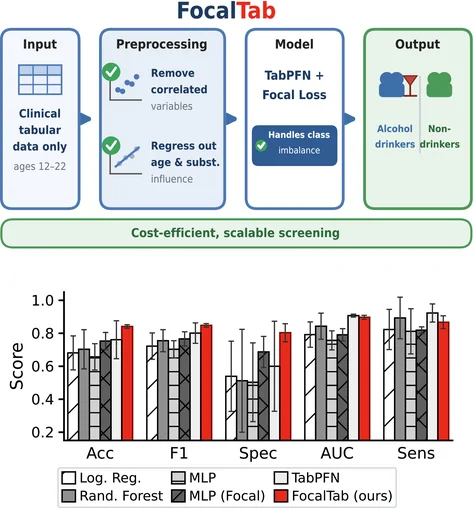

## 1 Introduction

Underage drinking refers to alcohol drinking among youth under 21 years of age. According to recent reports from the Substance Abuse and Mental Health Services Administration [1], over 30% of US adolescents have consumed alcohol at least once, and approximately 25% of these individuals engage in binge drinking [2]. Adolescent binge drinking is linked to both short‐ and long‐term adverse outcomes, including poor academic performance, increased accident risk, mental health disorders, violence, liver complications and a higher risk of future alcohol use disorder (AUD) [3]. These consequences also impose substantial societal costs, including increased healthcare spending, public safety burdens and productivity losses. Therefore, developing accurate classification models to identify adolescent drinkers' initiation and understanding the key variables that drive this transition are critical for enabling early identification and targeted intervention, which can significantly reduce the long‐term risk of alcohol dependence and related health consequences [4].

Machine learning (ML) models have shown considerable promise as early predictors and classifiers across clinical domains, including the derivation of imaging‐ and biomarker‐based indices for neurodegenerative and psychiatric conditions [5]. Building on this broader progress in clinical prediction, ML methods have been increasingly applied to classify alcohol use in adults [6, 7]; their application to adolescent populations remains underexplored. Among adolescent‐focused studies, most have relied on neuroimaging data and have only focused on a specific, relatively short age range. Whelan et al. [8] applied an elastic net to multimodal data, including structural magnetic resonance imaging (MRI), task‐based functional magnetic resonance imaging (fMRI) and personality, cognitive, environmental and genetic measures, from 692 adolescents aged 14 years in the IMAGEN study to classify current binge drinkers from non‐binge drinkers, achieving an accuracy of 91%. In practice, these imaging data are often inaccessible for many adolescents, as high acquisition costs and the requirement to live near an MRI facility impose significant financial and geographical barriers, restricting the diversity and representativeness of study samples. In contrast, clinical measurements (demographics, interview data, etc.) provide a more accessible and scalable alternative for studying alcohol consumption, enabling larger and more inclusive cohorts, which is essential for achieving higher accuracy and better performance.

Many prior studies on adolescent drinker detection [9, 10, 11] have not explicitly addressed age bias, leaving their models vulnerable to age‐driven classification. Age represents a major source of bias [12], as alcohol use increases with age. In addition, several clinical measures are themselves correlated with age, imposing an indirect source of bias. For example, studies in adolescents [13, 14] have shown that increasing age is associated with continued improvement in working memory capacity. Steinberg et al. [15] reported that both impulsivity and the effectiveness of parental monitoring decrease with increasing age among adolescents [16]. Laursen and Veenstra [17] noted that peer conformity peaks during early adolescence. Consequently, models trained on age‐related inputs may inadvertently learn age‐dependent patterns rather than alcohol‐specific signals.

Many studies [9, 18] used different types of substance use (cigarettes, cannabis, etc.) as classification variables for alcohol drinking classification, ignoring substance use bias. These variables are strongly associated with alcohol use, where alcohol often represents an early initiating substance that precedes later drug experimentation [19]. Incorporating these variables as predictors may artificially inflate model performance without capturing the underlying behavioural or psychosocial risk factors that independently contribute to alcohol misuse. Furthermore, many adolescents are not exposed to cigarettes or other substances [20], resulting in substantial class imbalance. This trend is supported by national data showing that, among adolescents aged 12–17, the prevalence of cigarette [21], e‐cigarette [22] and marijuana use all declined significantly in recent years [23]. In addition, even when adolescents did use substances, self‐report measures were subject to significant underreporting due to social desirability bias, particularly for sensitive and stigmatized behaviours [24].

Class imbalance represents another major challenge in adolescent alcohol‐drinking classification, as datasets typically contain substantially more non‐drinkers than drinkers [1, 2]. To address this issue, prior studies have employed strategies such as SMOTE‐based oversampling [10, 25], majority‐class undersampling [26] or extensive cross‐validation procedures [9]. However, these methods have inherent limitations: (1) SMOTE may generate synthetic samples that do not accurately reflect the minority‐class distribution, potentially introducing noise and overfitting; (2) undersampling discards informative majority‐class observations, reducing statistical power; and (3) repeated cross‐validation does not fundamentally correct the imbalance during model training. In contrast, focal loss [27] offers a more principled alternative by down‐weighting the contribution of majority‐class samples and emphasizing minority‐class examples during training, thereby enabling more effective use of the available data and improving classification performance under imbalanced settings.

**FIGURE 1:**
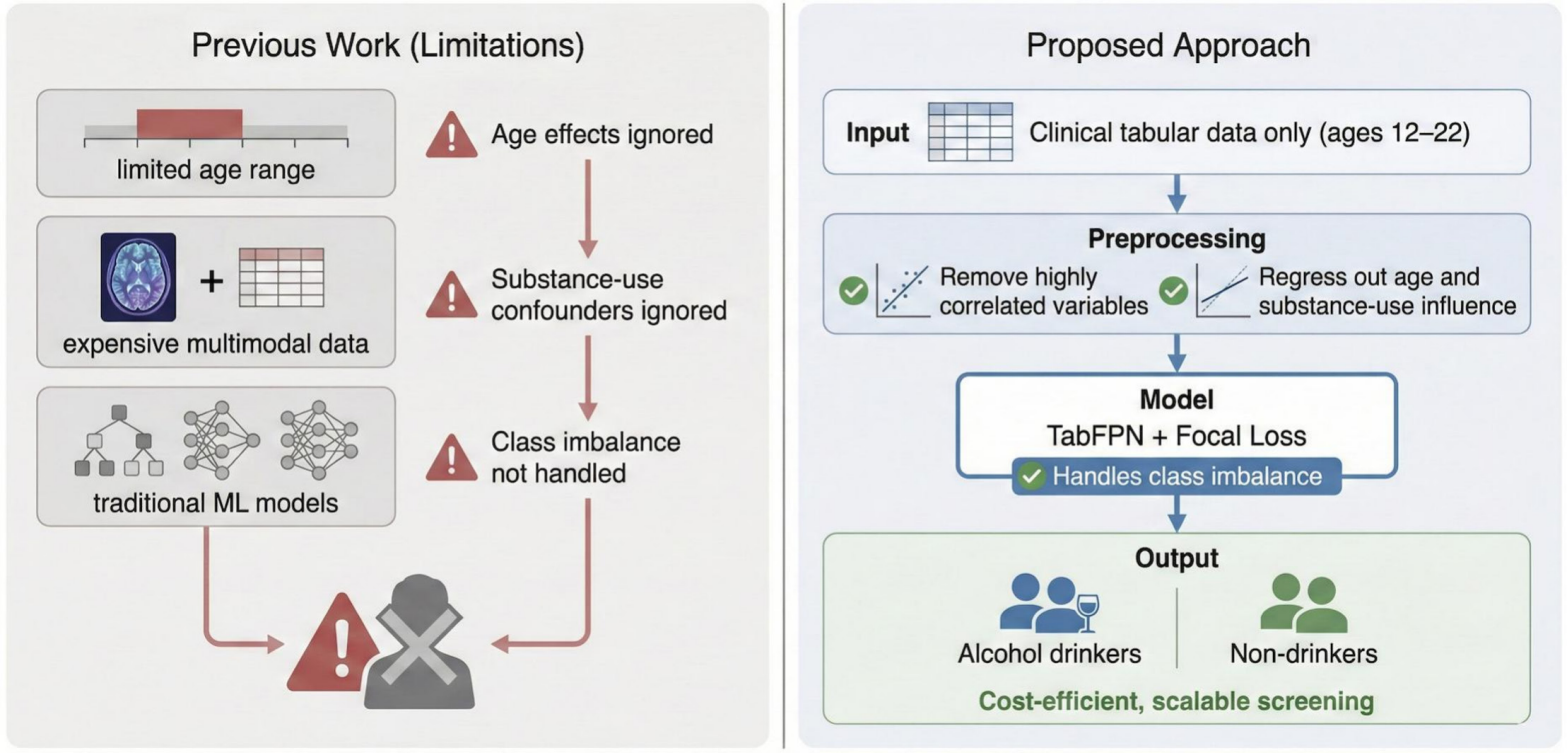
Comparison between prior adolescent alcohol use detection approaches and the proposed framework.

As illustrated in Figure 1, our proposed work addresses these methodological gaps through several key contributions. First, we develop a classification framework to detect adolescent drinking utilizing exclusively clinical measurements, enhancing accessibility and scalability for real‐world screening applications. Second, we expand our subjects' age range from 12 to 21 years, encompassing early adolescence through young adulthood, thereby including substantially more subjects while capturing the full trajectory of adolescent neurodevelopment. Third, we employ confound regression to remove age‐related variance from predictor variables, addressing the developmental biases that have limited prior work. Fourth, we construct a methodologically rigorous learning system that explicitly excludes substance use variables from the variable set, thereby eliminating potential data leakage and the confounding relations between alcohol and substance use and reducing the dependence on substance use. Finally, we incorporate focal loss to address the inherent class imbalance in adolescent drinking data, preserving maximum sample utilization while avoiding the distributional distortions introduced by synthetic oversampling. Collectively, these methodological innovations yield a reliable, generalizable classification system for distinguishing drinkers from non‐drinkers that addresses the key limitations of existing approaches in this field.

## 2 Materials and Methods

### 2.1 Participants

This study used data from the National Consortium on Alcohol and Neurodevelopment in Adolescence (NCANDA), a multi‐site longitudinal study designed to examine the effects of alcohol exposure on adolescent neurodevelopment [28]. Using an accelerated longitudinal design, NCANDA initially enrolled a sample of 831 adolescents aged 12–21 and conducted annual follow‐up assessments. The baseline data were analysed in this study to classify non‐rinkers/lower drinkers (shortened as non‐drinkers) versus moderate/heavy drinkers (shortened as drinkers) status using ML approaches.

Drinking status was defined following the modified Cahalan inventory [29] applied to the Customary Drinking and Drug Use Record [30]: Non‐drinkers reported minimal, infrequent use, drinking less than once per month, no more than three drinks per month and no binge episodes, whereas drinkers comprised the moderate and heavy categories, characterized by more regular drinking and/or higher per‐occasion quantities (e.g., roughly weekly drinking, or two or more drinks per occasion). Full classification criteria are in Data S1. Participants. There were 9 participants without baseline data and 21 participants without any follow‐up data, excluded from analyses. Based on these definitions, the final 801 samples included 661 non‐drinkers and 140 drinkers.

### 2.2 Variable Selection

To ensure the reliability and independence of input variables for ML, we applied the following variable selection criteria: (1) removal of constant variables that showed no variability across participants; (2) exclusion of variables with > 50% missing values; and (3) elimination of variables directly related to substance use, to prevent confounding or circularity in predicting drinking behaviour. After applying these criteria, a total of 167 baseline variables across behavioural, biological and environmental domains were retained for model development. Details can be found in Data S2 and Table 1.

**TABLE 1:** Variable categories and measurement instruments [1].

Category	*N* variables	Primary instruments	Key domains
Alcohol expectancy	7	AEQ [2]	Positive/negative beliefs about alcohol effects
Family history	4	FHAM [3]	Familial substance use disorders
Socio‐economic status	4	Demographic Interview, SES [4]	Education, income, occupation
Personality	5	TIPI [5], UPPS‐P [6]	Big Five traits, impulsivity
Environment	48	Multiple [7, 8, 9]	Neighbourhood, family, peer, school contexts
Child behaviour	21	Sleep Questionnaire [10, 11, 12], BRIEF [13], RSQ [14]	Sleep, executive function, coping
Medical history	5	TBI‐ID [15], Health Survey	Injuries, medical conditions
Demographics	5	Demographic Interview [1]	Family, education, activities
Anthropometrics	3	Physical Measures [1]	Height, weight, BMI
Pubertal development	2	PDS [16]	Physical maturation stage
Peer relationships	1	SSQ [17]	Social support quality
Daily routine	31	Multiple [1, 18, 19, 20]	Activities, competence, functioning
Psychiatric symptoms	31	SSAGA [21], CES‐D [22]	Depression, conduct, ADHD, PTSD, OCD, anxiety, panic
**Total**	**167**		

### 2.3 Classification of Drinkers

**FIGURE 2:**
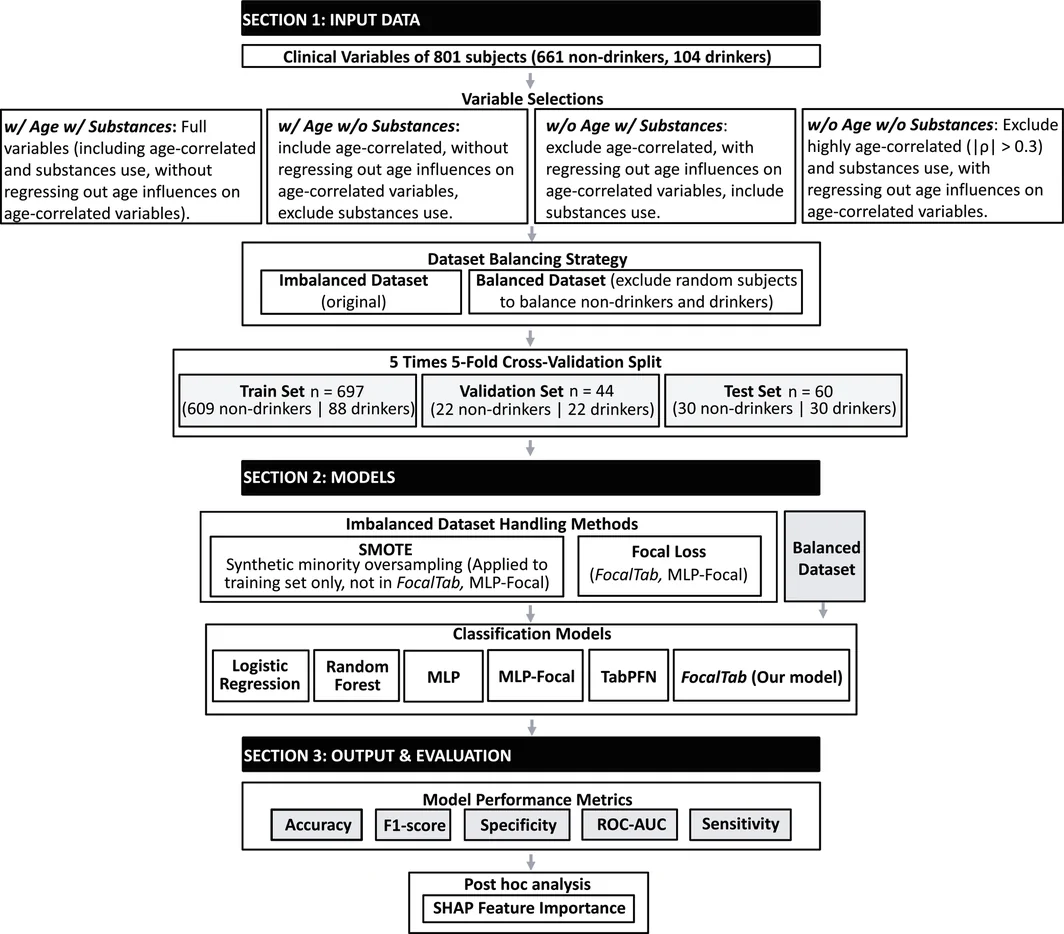
Pipeline of data preprocessing, model development and evaluation procedures.

We developed an ML model to classify adolescent drinkers versus non‐drinkers using baseline clinical data from the NCANDA dataset. The overall workflow is summarized in Figure 2, which illustrates the data preprocessing strategies, model development and evaluation procedures. Briefly, multiple input variable configurations were constructed to explicitly address potential confounding effects of age and substance use variables, followed by strategies to handle class imbalance. The processed data were then used to train and evaluate classification models.

Among the evaluated models, we implemented **TabPFN (Tabular Prior‐Data Fitted Network)** [31] with **focal loss** [27] (*FocalTab*) as the primary model, given its strong performance on tabular clinical data and its ability to improve minority‐class learning. The detailed methodology is described in the following subsections: Section 2.3.1 outlines the model development pipeline, including input variable configurations, data preprocessing and the TabPFN architecture with focal loss; Section 2.3.2 describes the training strategy, including dataset balancing and cross‐validation; and Section 2.3.3 presents the evaluation framework, including comparisons with other classification models, approaches for handling class imbalance and strategies for mitigating age‐ and substance use‐related bias. Post hoc analyses were subsequently conducted to enhance model interpretability and biological relevance, with variable importance analysis described in Section 2.3.4.

#### 2.3.1 Model Development via *FocalTab*

In the preprocessing step, missing values were imputed using medians in the training set. Meanwhile, we excluded substance use‐related variables, regressed out age effects and removed variables highly correlated with age to mitigate age‐related bias (see Section 2.3.3 for details). The resulting variable set was then used as input to the *FocalTab* model to classify participants as drinkers versus non‐drinkers.

TabPFN [31] is a pretrained, transformer‐based classification foundation model designed for tabular classification via in‐context learning. Unlike conventional ML methods that require iterative training on each new dataset, TabPFN leverages knowledge acquired during an offline pretraining phase on large collections of synthetic datasets. The architecture uses alternating row and column attention mechanisms and approximates Bayesian inference in a single forward pass.

Standard TabPFN performs in‐context inference with its pretrained weights frozen. In FocalTab, we instead fine‐tuned the model: The pretrained transformer weights were unfrozen and updated by standard gradient descent on the training data. For each batch, a forward pass produced the model's predictions, the focal loss was computed, back‐propagation produced the gradients, and an optimizer step updated the weights.

The model accepts training samples $D_{\text{train}}=\left\{\left(x_{1},y_{1}\right),\ldots ,\left(x_{n},y_{n}\right)\right\}$ and test variables $x_{t\mathrm{est}}$ as set‐valued inputs, yielding the posterior predictive distribution (PPD) $q_{\theta }\;\left(y\;\right|x_{\text{test}},D_{\text{train}})$ without requiring gradient‐based optimization on the target dataset [31]. The PPD for a test sample $x_{\text{test}}$ is approximated as:

(1) $$\begin{array}{c} q_{\theta }(y|x_{t\mathrm{est}},D_{\text{train}})\approx p(y|x_{t\mathrm{est}},D_{\text{train}})\propto \int \Phi p\left(y,x_{t\mathrm{est}}|\varphi \right)\;\\ p\left(D_{\text{train}}|\varphi \right)p\left(\varphi \right)d\varphi \end{array}$$

where φ represents hypotheses from a prior space Φ that includes structural causal models (SCMs) and Bayesian neural networks (BNNs), weighted by their likelihood given the training data and prior probability [31].

To address the substantial class imbalance in our dataset (661 non‐drinkers vs. 140 drinkers), we employed focal loss [27] as the model's loss function, which down‐weights the contribution of easily classified examples and focuses training on difficult, misclassified samples. The focal loss is defined as follows:

(2) $$\mathrm{FL}\left(p_{t}\right)=-\alpha_{t}\;{\left(1-p_{t}\right)}^{\gamma }\;\log\left(p_{t}\right)$$

where $\alpha_{t}$ and γ are the parameters to be optimized, while $p_{t}$ represents the model's estimated probability for the ground‐truth class:

(3) $$p_{t}=\left\{\;p\;\text{if}\;y=1;1-p_{\text{otherwise}}\right\}$$

#### 2.3.2 Training Strategy

The TabPFN model was optimized using a multistaged approach designed to maximize predictive performance while rigorously segregating training and evaluation data. Initially, we conducted an extensive grid search to identify optimal hyperparameters, fine‐tuning the model on the training set across a spectrum of learning rates (5 × 10^−6^ to 1 × 10^−2^) and the RAdam optimizer. To address potential class imbalance, focal loss was employed, with hyperparameters γ and α tuned via incremental intervals on the validation set. The optimal classification threshold and hyperparameter configuration were determined by selecting the settings that yielded the highest F1‐score and AUC during validation. Subsequently, the model was retrained on the combined training and validation cohorts using the previously identified optimal settings to ensure robust variable representation. Finally, the performance of the refitted model was assessed on a strictly held‐out test set, with all classification thresholds fixed from the validation phase to ensure an unbiased evaluation of the model's generalizability. The other baseline models' experiment protocol can be found in Data S3.

#### 2.3.3 Evaluation

We evaluated our model across three dimensions: (1) comparison of model performance with other state‐of‐the‐art methods using 5 times 5‐fold cross‐validation (CV), (2) assessment of the impact of class imbalance strategies and (3) examination of substance use and age biases in classifying adolescent drinkers from non‐drinkers.

To further assess the performance of our model, we compared it with other methods, including (1) logistic regression [32], (2) random forest [33], (3) multilayer perceptron (MLP) [34] and (4) TabPFN without focal loss. For the MLP, we implemented two variants: MLP with default binary cross‐entropy loss (MLP) [35] and MLP with focal loss (MLP‐Focal). Model performance was evaluated using 5 times 5‐fold CV. In each fold, subjects were randomly divided into training, validation, and test sets. The training set contained 697 subjects (609 non‐drinkers and 88 drinkers), the validation set contained 44 subjects (22 non‐drinkers and 22 drinkers), and the test set contained 60 subjects (30 non‐drinkers and 30 drinkers). Average accuracy, F1‐score, specificity and AUC were calculated across the 5 times 5‐fold CV results to evaluate each model.

To evaluate the impact of class imbalance on model performance, we compared four sampling strategies: (1) ***Imbalanced‐Original***, using the original class distribution; (2) **Focal loss**, applying focal loss to *Imbalanced‐Original*; (3) ***Imbalanced‐SMOTE***, using the Synthetic Minority Over‐sampling Technique 30 to generate synthetic drinkers samples equal to the non‐drinkers' class size; and (4) ***Balanced***, randomly downsampling non‐drinker subjects in the training set to match the drinkers count (*n* = 88 per group). Validation and test sets were kept identical across strategies to ensure comparability.

To evaluate the impact of substance use variables on classification of drinkers versus non‐drinkers, we compared four variable selection strategies: (1) *w/ Age w/ Substances*, all variables included, comprising substance use variables and highly age‐correlated variables, without regressing out age effects; (2) *w/o Age w/o Substances*, substance use variables and highly age‐correlated variables excluded, with age effects regressed out from age‐correlated variables; (3) *w/Age w/o Substances*, substance use variables excluded while highly age‐correlated variables retained, without regressing out age effects; and (4) *w/o Age w/Substances*, substance use variables retained while highly age‐correlated variables excluded, with age effects regressed out from age‐correlated variables.

To mitigate age‐related confounding effects on classification performance, we implemented a multistage variable filtering strategy. Variables with strong age correlations (|ρ| > 0.3; FDR‐corrected) were excluded. Variables with moderate but significant correlations (q < 0.05 after FDR correction) were residualized using linear regression: numeric variables via ordinary least squares, $x_{\text{residual}}=x-\left(\beta_{0}+\beta_{1}\cdot \mathrm{age}\right)$, and binary variables via logistic regression residuals, $x_{\text{residual}}=x-\hat{p}\left(x=1|\mathrm{age}\right)$. Because residualization is applied only to variables with moderate (not strong) age dependence and over the relatively narrow adolescent age range of the sample, a linear/logistic specification captures the dominant age‐related variance while avoiding the overfitting that higher‐order per‐variable age models would incur given the limited number of positive cases. The age variable was then removed from the variable matrix. Remaining numeric variables were standardized using parameters derived from the training set only.

#### 2.3.4 Model Interpretation With SHAP

SHapley Additive exPlanations (SHAP) [36] is a widely used framework for interpreting ML models by quantifying the contribution of individual variables to model prediction. In this study, we applied SHAP to interpret the *FocalTab* model and identify the most influential variables for drinkers' classification. Specifically, SHAP values were computed for all input variables to estimate their marginal contributions to the predicted drinking outcomes. Variables were subsequently ranked according to their mean absolute SHAP values, enabling identification of the most important predictors driving model performance.

## 3 Results

### 3.1 Demographics Analysis

As shown in Table 2, drinker subjects (18.55 ± 1.98 years) were significantly older than non‐drinker subjects (15.65 ± 2.31 years; *p* < 0.001). There was no significant difference in gender distribution between groups (*p* = 0.200). Racial composition was similar across groups, with European‐American participants comprising the majority in both the non‐drinkers (70.8%) and drinkers (76.4%) groups; no significant differences were observed in any racial category among each group (all *p* > 0.1).

**TABLE 2:** Demographic characteristics of non‐drinker and drinker groups.

Variable	Non‐drinkers	Drinkers	*p*
*N*	661	140	
Age (in years)	15.65 ± 2.31	18.55 ± 1.98	< 0.001***
Gender			
Female	326 (49.3%)	78 (55.7%)	0.199
Male	335 (50.7%)	62 (44.3%)	
Race			
Native American	3 (0.5%)	0 (0.0%)	0.578
Asian	50 (7.6%)	11 (7.9%)	
Pacific Islander	3 (0.5%)	1 (0.7%)	
African‐American	78 (11.8%)	14 (10.0%)	
European‐American	468 (70.8%)	107 (76.4%)	
Other	59 (8.9%)	7 (5.0%)	

*Note:* Values are presented as mean ± standard deviation for continuous variables (age) and count (percentage) for categorical variables (gender and race). Statistical comparisons between groups were conducted using independent‐samples *t*‐tests for age and chi‐square tests for categorical variables of gender and race. Significance levels: *p < 0.05, **p < 0.01, ***p < 0.001.

### 3.2 Comparison of Classification Performance With Different Models

#### 3.2.1 Model Comparison

**FIGURE 3:**
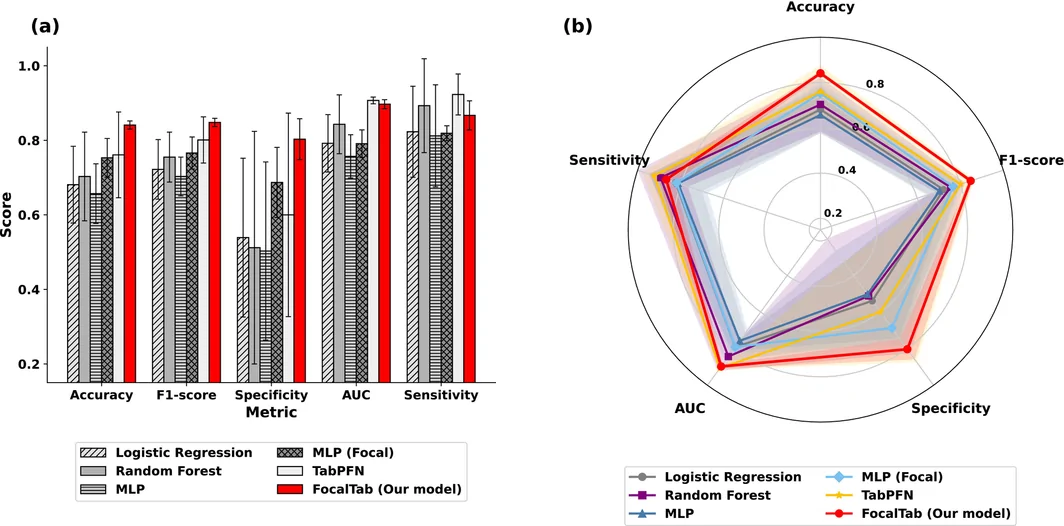
Model performance comparison for drinkers' classification using the *Imbalanced‐Original* dataset with the *w/o Age w/o Substances* variable setting. (a) Bar plot of performance metrics across models; *FocalTab* is highlighted in red. (b) Radar plot of performance metrics across models; *FocalTab* is highlighted in red. AUC, ROC‐AUC; MLP, multilayer perceptron.

Table 3 and Figure 3 presented comparisons of six models' performance for classifying drinkers versus non‐drinkers using the *Imbalanced‐Original* dataset with the *w/o Age w/o Substances* variable setting. *FocalTab* demonstrated the most robust performance under strict confound control, achieving an AUC of 0.897 ± 0.012, accuracy of 0.841 ± 0.011, F1‐score of 0.848 ± 0.011, a sensitivity of 0.867 ± 0.039 and a specificity of 0.803 ± 0.055 (Table 3). Notably, the specificity of all other models was substantially lower than that of *FocalTab* under these settings.

**TABLE 3:** Comparison of model performance for drinkers' classification using the *Imbalanced‐Original* dataset with the *w/o Age w/o Substances* variable setting.

Metric	Logistic regression	Random forest	MLP	MLP (Focal)	TabPFN	*FocalTab* (our model)
Accuracy	0.681 ± 0.103	0.703 ± 0.119	0.657 ± 0.080	0.753 ± 0.052	0.761 ± 0.115	**0.841 ± 0.011**
F1‐score	0.722 ± 0.080	0.755 ± 0.067	0.703 ± 0.052	0.766 ± 0.043	0.801 ± 0.062	**0.848 ± 0.011**
Specificity	0.539 ± 0.213	0.512 ± 0.312	0.503 ± 0.239	0.687 ± 0.094	0.600 ± 0.273	**0.803 ± 0.055**
AUC	0.792 ± 0.077	0.843 ± 0.079	0.757 ± 0.058	0.791 ± 0.037	**0.907 ± 0.009**	0.897 ± 0.012
Sensitivity	0.823 ± 0.122	0.893 ± 0.126	0.812 ± 0.137	0.819 ± 0.020	**0.923 ± 0.055**	0.867 ± 0.039

*Note:* Bold: *FocalTab*.

Abbreviations: AUC, ROC‐AUC; MLP, multilayer perceptron.

#### 3.2.2 Substance Use and Age Biases

**FIGURE 4:**
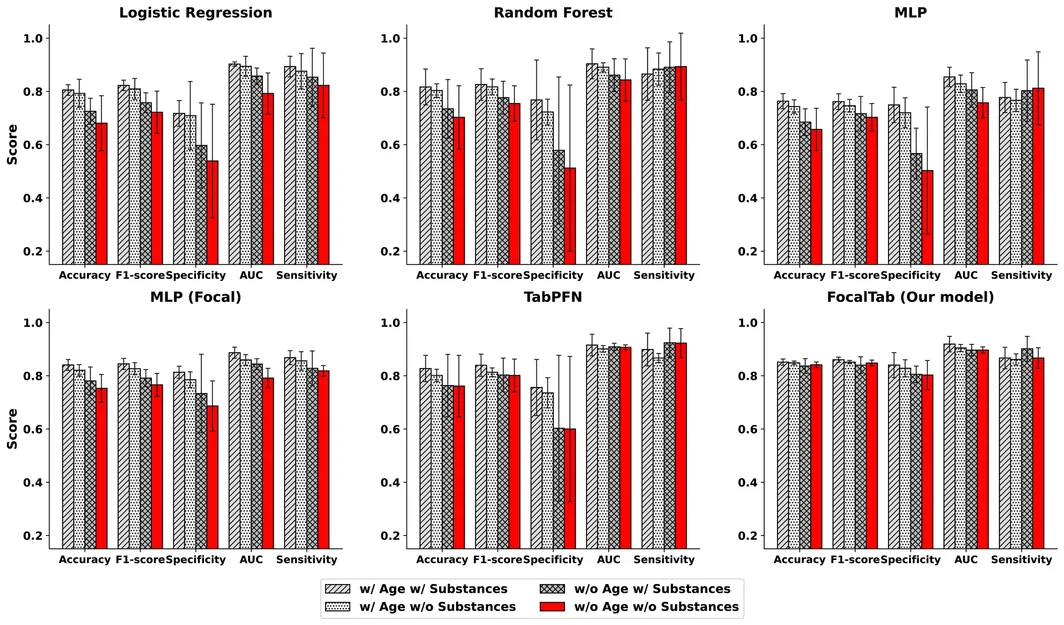
Bar plots of model performance across variable selection strategies for evaluating substance use and age biases using the *Imbalanced‐Original* dataset setting. From left to right, top to bottom: Logistic regression, random forest, MLP, MLP‐Focal, TabPFN and *FocalTab*. AUC, ROC‐AUC; MLP, multilayer perceptron.

**FIGURE 5:**
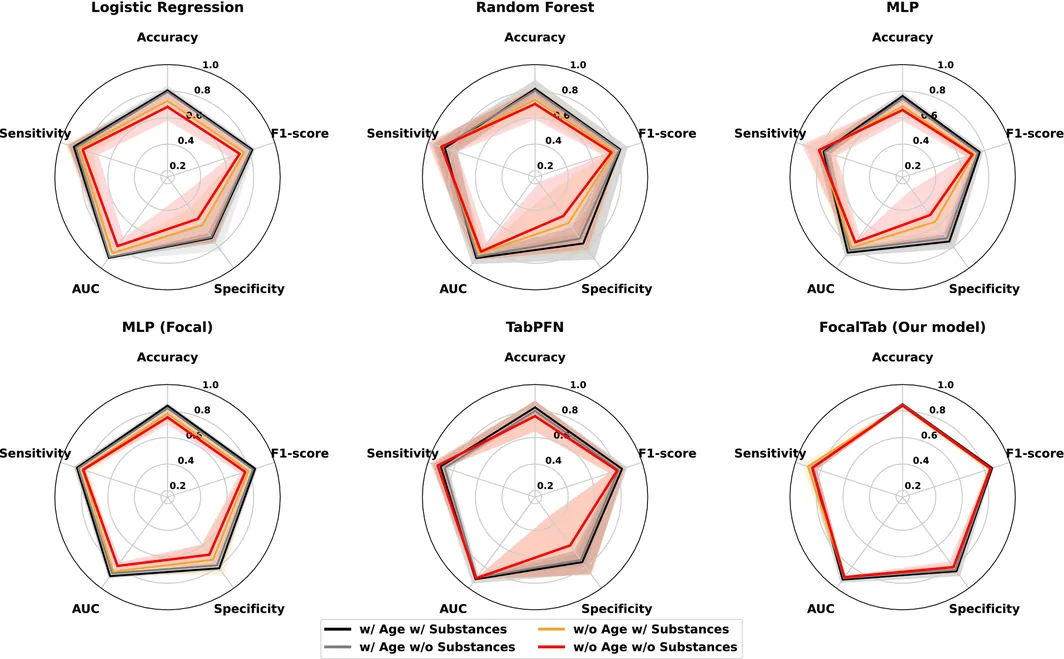
Radar plots of model performance across variable selection strategies for evaluating substance use and age biases using the *Imbalanced‐Original* dataset setting. From left to right, top to bottom: Logistic regression, random forest, MLP, MLP‐Focal, TabPFN and *FocalTab*. AUC, ROC‐AUC; MLP, multilayer perceptron.

Table 4 and Figures 4 and 5 summarized the performance of six models in classifying drinkers versus non‐drinkers under the *Imbalanced‐Original* dataset, highlighting the effects of substance use and age biases. Across models, performance generally decreased as biases were removed, indicating that many classifiers relied on age‐related developmental variance and/or substance use variables to achieve high accuracy.

**TABLE 4:** Comparison of model performance across variable selection strategies for evaluating substance use and age biases using the *Imbalanced‐Original* dataset setting.

Dataset setting	Metric	Logistic regression	Random forest	MLP	MLP (Focal)	TabPFN	*FocalTab* (our model)
*w/Age w/Substances*	Accuracy	0.805 ± 0.020	0.817 ± 0.067	0.763 ± 0.028	0.841 ± 0.020	0.827 ± 0.049	**0.851 ± 0.012**
	F1‐score	0.823 ± 0.020	0.826 ± 0.059	0.762 ± 0.029	0.845 ± 0.021	0.840 ± 0.042	**0.860 ± 0.010**
	Specificity	0.717 ± 0.048	0.768 ± 0.150	0.749 ± 0.066	0.813 ± 0.023	0.756 ± 0.105	**0.840 ± 0.047**
	AUC	0.903 ± 0.008	0.904 ± 0.056	0.854 ± 0.036	0.887 ± 0.021	0.915 ± 0.041	**0.919 ± 0.029**
	Sensitivity	0.893 ± 0.039	0.865 ± 0.099	0.777 ± 0.057	0.868 ± 0.026	**0.899 ± 0.062**	0.867 ± 0.040
*w/Age w/o Substances*	Accuracy	0.793 ± 0.053	0.803 ± 0.025	0.743 ± 0.025	0.821 ± 0.021	0.801 ± 0.023	**0.849 ± 0.007**
	F1‐score	0.810 ± 0.039	0.817 ± 0.030	0.746 ± 0.024	0.827 ± 0.023	0.813 ± 0.016	**0.851 ± 0.006**
	Specificity	0.709 ± 0.128	0.723 ± 0.048	0.720 ± 0.056	0.785 ± 0.030	0.736 ± 0.057	**0.828 ± 0.032**
	AUC	0.894 ± 0.038	0.891 ± 0.017	0.829 ± 0.033	0.860 ± 0.020	0.901 ± 0.012	**0.905 ± 0.013**
	Sensitivity	0.876 ± 0.066	**0.884 ± 0.061**	0.767 ± 0.042	0.856 ± 0.035	0.867 ± 0.017	0.861 ± 0.021
*w/o Age w/Substances*	Accuracy	0.725 ± 0.049	0.735 ± 0.110	0.685 ± 0.050	0.781 ± 0.052	0.763 ± 0.117	**0.837 ± 0.028**
	F1‐score	0.758 ± 0.037	0.776 ± 0.062	0.716 ± 0.065	0.791 ± 0.032	0.803 ± 0.063	**0.840 ± 0.031**
	Specificity	0.597 ± 0.160	0.579 ± 0.276	0.567 ± 0.095	0.733 ± 0.147	0.603 ± 0.274	**0.805 ± 0.031**
	AUC	0.857 ± 0.030	0.861 ± 0.062	0.806 ± 0.065	0.844 ± 0.020	**0.909 ± 0.013**	0.896 ± 0.022
	Sensitivity	0.853 ± 0.109	0.891 ± 0.096	0.803 ± 0.115	0.828 ± 0.065	**0.924 ± 0.055**	0.901 ± 0.047
*w/o Age w/o Substances*	Accuracy	0.681 ± 0.103	0.703 ± 0.119	0.657 ± 0.080	0.753 ± 0.052	0.761 ± 0.115	**0.841 ± 0.011**
	F1‐score	0.722 ± 0.080	0.755 ± 0.067	0.703 ± 0.052	0.766 ± 0.043	0.801 ± 0.062	**0.848 ± 0.011**
	Specificity	0.539 ± 0.213	0.512 ± 0.312	0.503 ± 0.239	0.687 ± 0.094	0.600 ± 0.273	**0.803 ± 0.055**
	AUC	0.792 ± 0.077	0.843 ± 0.079	0.757 ± 0.058	0.791 ± 0.037	**0.907 ± 0.009**	0.897 ± 0.012
	Sensitivity	0.823 ± 0.122	0.893 ± 0.126	0.812 ± 0.137	0.819 ± 0.020	**0.923 ± 0.055**	0.867 ± 0.039

*Note:* Bold: *FocalTab*.

Abbreviations: AUC, ROC‐AUC; MLP, multilayer perceptron.

Figure 4 illustrated a consistent performance ordering across the four variable conditions: *w/ Age w/ Substances* (highest) > *w/ Age w/o Substances* > *w/o Age w/ Substances* > *w/o Age w/o Substances* (lowest). The decline was most pronounced for specificity, suggesting that removing the age and substance use confounds primarily reduced the models' ability to identify non‐drinkers correctly. Specifically, specificity fell sharply from the *w/Age w/Substances* to *w/o Age w/o Substances* conditions for logistic regression (0.717 ± 0.048 to 0.539 ± 0.213), random forest (0.768 ± 0.150 to 0.512 ± 0.312), MLP (0.749 ± 0.066 to 0.503 ± 0.239), MLP with focal loss (0.813 ± 0.023 to 0.687 ± 0.094) and TabPFN (0.756 ± 0.105 to 0.600 ± 0.273; Table 4). This pattern indicated that, once age and substance use biases were controlled, conventional models misclassified non‐drinkers at substantially higher rates.

As shown in Table 4, *w/ Age w/o Substances* (retaining age‐correlated variables without age regression) typically outperformed *w/o Age w/ Substances* (retaining substance‐use variables while regressing out age effects), suggesting that developmental variance contributed more strongly to model discrimination than substance‐use variables in this dataset. For example, random forest achieved AUC = 0.891 ± 0.017 under *w/Age w/o Substances*, but dropped to AUC = 0.861 ± 0.062 under *w/o Age w/Substances*, using the *Imbalanced‐Original* setting (Table 4). Similar trends were observed across other models, consistent with age‐linked variables serving as a major driver of classification separability.

#### 3.2.3 Class Imbalance

**FIGURE 6:**
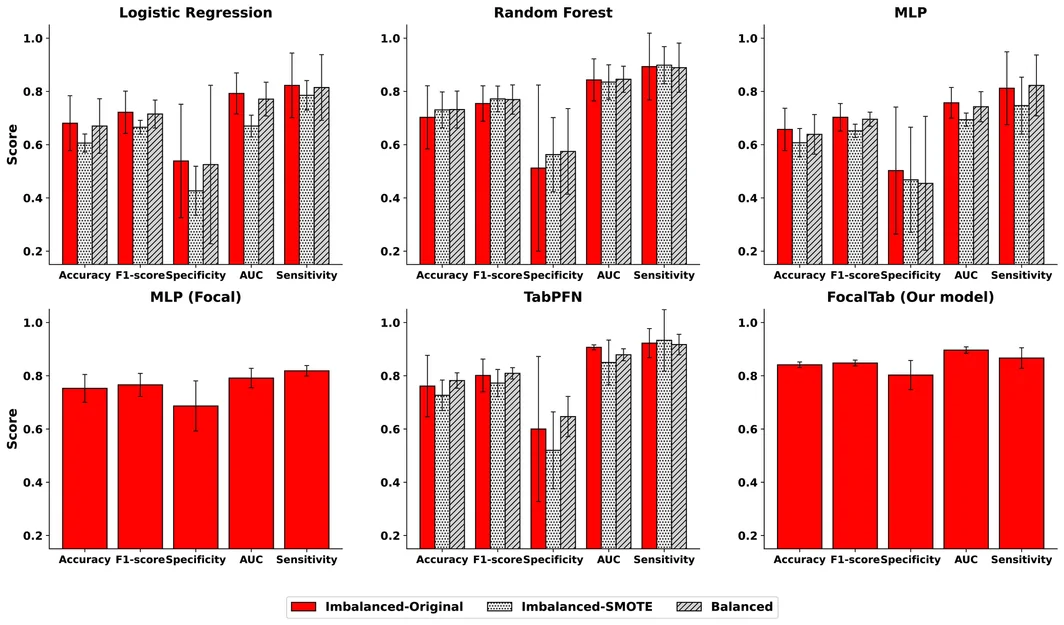
Bar plots of model performance across class imbalance strategies using the *Exclude* variable setting. From left to right, top to bottom: Logistic regression, random forest, MLP, MLP‐Focal, TabPFN and *FocalTab Note*: AUC, ROC‐AUC; MLP, multilayer perceptron.

**FIGURE 7:**
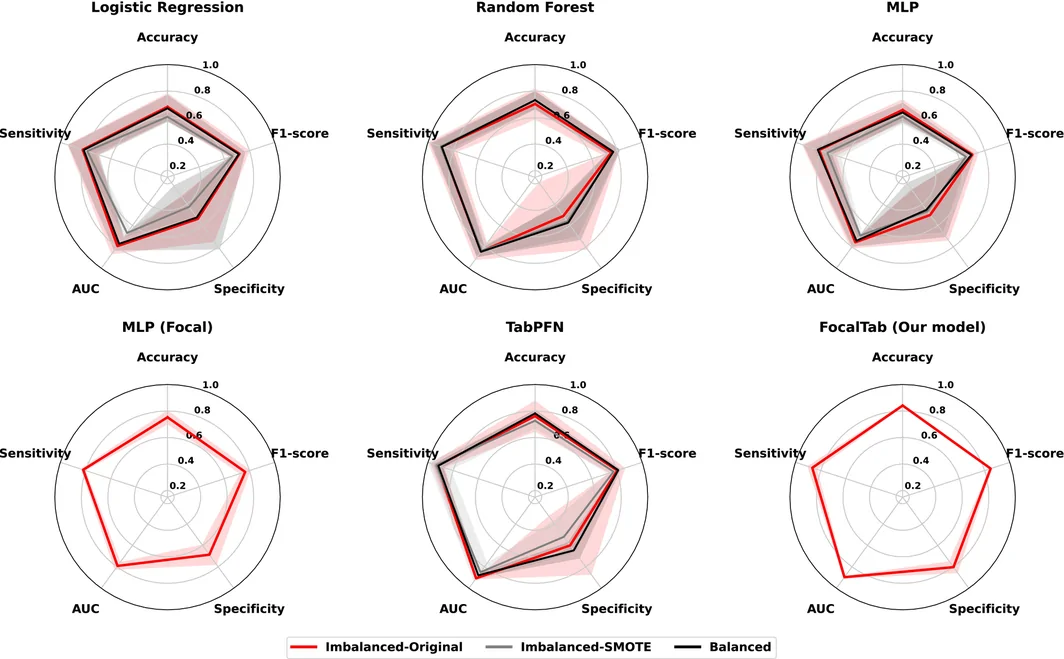
Radar plots of model performance across class imbalance strategies using the *w/o Age w/o Substances* variable setting. From left to right, top to bottom: Logistic regression, random forest, MLP, MLP‐Focal, TabPFN and *FocalTab.* AUC, ROC‐AUC; MLP, multilayer perceptron.

Table 5 and Figures 6 and 7 presented comparisons of six models' performance for classifying drinkers versus non‐drinkers using the *w/o Age w/o Substances* variable setting, revealing the effects of class imbalance on classification. Model performance under the *Balanced* setting was slightly better than under the two *Imbalanced* settings. Under the *Imbalanced‐SMOTE* and *Balanced* settings, where *FocalTab* was not evaluated, TabPFN achieved the highest AUCs (0.850 ± 0.084 with *Imbalanced‐SMOTE*; 0.879 ± 0.022 with *Balanced*); however, specificity remained substantially lower (0.520 ± 0.145 and 0.647 ± 0.076, respectively) than *FocalTab's* specificity of 0.803 ± 0.055 on the *Imbalanced‐Original* setting (Table 5). Notably, *FocalTab* demonstrated the most robust performance in the *Imbalanced‐Original* setting, achieving an AUC of 0.897 ± 0.012, an accuracy of 0.841 ± 0.011, an F1‐score of 0.848 ± 0.011, a sensitivity of 0.867 ± 0.039 and a specificity of 0.803 ± 0.055 (Table 5). This specificity substantially exceeded that of all competing methods under the same *w/o Age w/o Substances* condition, indicating that *FocalTab* could correctly identify non‐drinkers even on highly imbalanced datasets.

**TABLE 5:** Comparison of model performance across class imbalance strategies using the *w/o Age w/o Substances* variable setting.

Dataset setting	Metric	Logistic regression	Random forest	MLP	MLP (focal)	TabPFN	*FocalTab* (our model)
*Imbalanced‐Original*	Accuracy	0.6807 ± 0.1031	0.703 ± 0.119	0.657 ± 0.080	0.753 ± 0.052	0.761 ± 0.115	**0.841 ± 0.011**
	F1‐score	0.7216 ± 0.0795	0.755 ± 0.066	0.703 ± 0.052	0.766 ± 0.043	0.801 ± 0.062	**0.848 ± 0.011**
	Specificity	0.5387 ± 0.2131	0.512 ± 0.312	0.503 ± 0.239	0.687 ± 0.094	0.600 ± 0.273	**0.803 ± 0.055**
	AUC	0.7924 ± 0.0771	0.843 ± 0.079	0.757 ± 0.058	0.791 ± 0.037	**0.907 ± 0.009**	0.897 ± 0.012
	Sensitivity	0.8227 ± 0.1215	0.893 ± 0.126	0.812 ± 0.137	0.819 ± 0.020	**0.923 ± 0.055**	0.867 ± 0.039
*Imbalanced‐SMOTE*	Accuracy	0.6060 ± 0.0342	**0.731 ± 0.067**	0.607 ± 0.053	—	0.727 ± 0.057	—
	F1‐score	0.6654 ± 0.0260	0.772 ± 0.048	0.652 ± 0.025	—	**0.773 ± 0.051**	—
	Specificity	0.4267 ± 0.0922	**0.563 ± 0.139**	0.468 ± 0.198	—	0.520 ± 0.145	—
	AUC	0.6699 ± 0.0409	0.835 ± 0.065	0.694 ± 0.025	—	**0.850 ± 0.084**	—
	Sensitivity	0.7853 ± 0.0556	0.899 ± 0.070	0.747 ± 0.107	—	**0.933 ± 0.116**	—
*Balanced*	Accuracy	0.6700 ± 0.1026	0.732 ± 0.069	0.639 ± 0.075	—	**0.782 ± 0.029**	—
	F1‐score	0.7151 ± 0.0525	0.770 ± 0.055	0.696 ± 0.027	—	**0.809 ± 0.021**	—
	Specificity	0.5253 ± 0.2977	0.575 ± 0.161	0.455 ± 0.252	—	**0.647 ± 0.076**	—
	AUC	0.7711 ± 0.0635	0.846 ± 0.049	0.743 ± 0.057	—	**0.879 ± 0.022**	—
	Sensitivity	0.8147 ± 0.1234	0.889 ± 0.092	0.823 ± 0.114	—	**0.917 ± 0.039**	—

*Note:* Bold: *FocalTab*.

Abbreviations: AUC, ROC‐AUC; MLP, multilayer perceptron.

### 3.3 Model Interpretation With SHAP

**FIGURE 8:**
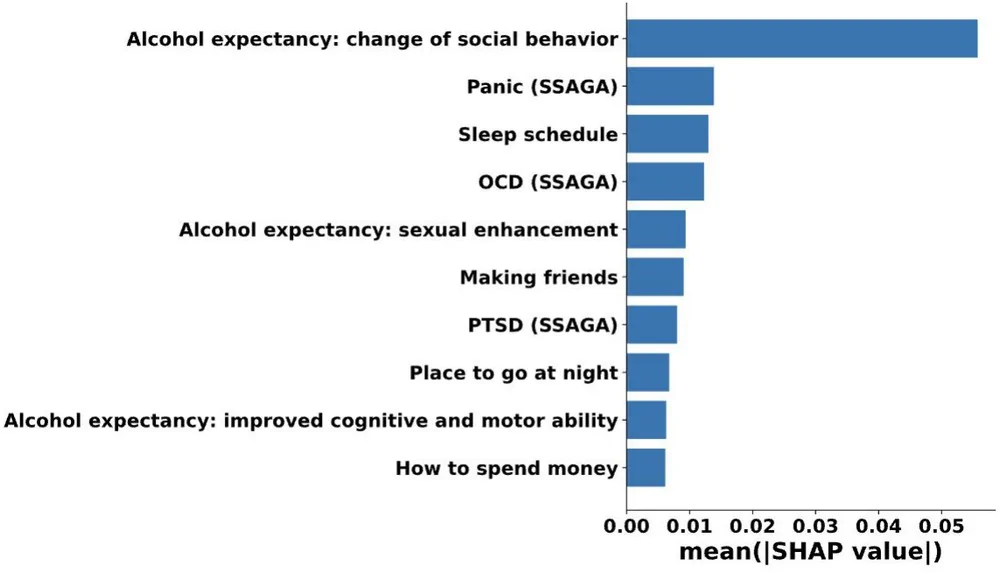
Top 10 most important variables ranked by mean absolute SHAP values from the *FocalTab* model for drinkers classification using the *Imbalanced‐Original* dataset with the *w/o Age w/o Substances* variable setting. *Note*: SSAGA refers to Semi‐Structured Assessment for the Genetics of Alcoholism.

Figure 8 presented the Top 10 most important variables with the highest mean absolute SHAP values from the *FocalTab* model for drinkers' classification using the *Imbalanced‐Original* dataset with the *w/o Age w/o Substances* variable setting. Higher values indicated greater variable importance. The most influential variables included alcohol expectancy (anticipated change of social behaviour, sexual enhancement, improved cognitive and motor ability), psychiatric symptoms (panic, OCD and PTSD), sleep schedule, making friends, place to go at night and how to spend money.

## 4 Discussion

In this study, we developed a novel TabPFN classification framework with focal loss for distinguishing adolescent drinkers from non‐drinkers using exclusively clinical variables obtained through routine medical encounters. Specifically, we utilize a broader adolescent age range (12–21 years), enabling comprehensive modelling across the full developmental trajectory from early adolescence through young adulthood. To mitigate age‐related bias, we explicitly account for age confounding through systematic confound regression. In addition, to prevent potential data leakage and inflated predictive performance, substance‐use variables are excluded from the variable set. Finally, severe class imbalance is addressed using focal loss rather than synthetic oversampling, thereby preserving the original data distribution while improving the model's ability to learn from minority‐class samples.

The superior performance of *FocalTab* under strict confound control, achieving AUC 0.897 ± 0.012, accuracy 0.841 ± 0.011 and specificity 0.803 ± 0.055 (Table 3), aligns with recent advances in tabular ML [31, 37]. Its Bayesian approximation properties and smooth decision boundaries make it particularly suited to clinical tasks with limited samples, and our dataset (*N* = 801) falls within its optimal operating range. When confounds were not fully removed, TabPFN achieved the strongest performance (e.g., AUC 0.879 ± 0.022, accuracy 0.782 ± 0.029 in the *Balanced w/o Age w/o Substances* setting; Table 5), though combining it with focal loss proved essential under the most stringent conditions.

Our focal loss implementation addressed the substantial class imbalance (661 non‐drinkers vs. 140 drinkers; ~5:1) at the algorithmic level, offering key advantages over data‐level approaches such as SMOTE. Although competing models on the same imbalanced data exhibited severe majority‐class bias, specificity ranging from 0.503 ± 0.239 to 0.687 ± 0.094 despite sensitivity of 0.812 ± 0.137 to 0.893 ± 0.126, *FocalTab* maintained a specificity of 0.803 ± 0.055 (Table 5). SMOTE yielded only modest specificity gains (e.g., logistic regression 0.539 ± 0.213 to 0.427 ± 0.092, random forest 0.512 ± 0.312 to 0.563 ± 0.139) that remained far below *FocalTab* and actually deteriorated TabPFN's specificity to 0.520 ± 0.145. Focal loss was originally developed for extreme foreground–background imbalance in object detection [38], dynamically down‐weights well‐classified examples to focus learning on hard samples. Prior work confirms its advantage: Baloch et al. [39] showed that their focal loss variants outperformed both SMOTE and near‐miss undersampling on 4 out of 6 highly imbalanced fraud detection datasets, achieving gains of up to 3% in balanced accuracy without the computational overhead of synthetic sample generation. In network intrusion detection systems, Mulyanto et al. [40] found that whereas SMOTE combined with deep neural networks achieved acceptable accuracy, F1‐scores remained inadequate due to persistent class imbalance issues that focal loss successfully addressed. Beyond performance, focal loss avoids SMOTE's known drawbacks, including synthetic samples that may misrepresent true distributions, increased class overlap near decision boundaries and noise amplification in high‐dimensional spaces [25, 41].

Performance across dataset settings followed a consistent ordering: *w/ Age w/ Substances* > *w/ Age w/o Substances* > *w/o Age w/ Substances* > *w/o Age w/o Substances*, indicating that competing models relied heavily on developmental variance and substance use information to achieve apparently high accuracy (Table 4). Specificity declined most sharply upon confound removal: Logistic regression fell from 0.717 ± 0.048 to 0.539 ± 0.213, random forest from 0.768 ± 0.150 to 0.512 ± 0.312, MLP from 0.749 ± 0.066 to 0.503 ± 0.239, MLP with focal loss from 0.813 ± 0.023 to 0.687 ± 0.094, and TabPFN from 0.756 ± 0.105 to 0.600 ± 0.273 (Table 4), meaning these models misclassified over 80% of non‐drinkers once confounds were controlled. The stronger impact of age removal relative to substance use removal (e.g., random forest AUC 0.891 ± 0.017 under *w/Age w/o Substances* vs. 0.861 ± 0.062 under *w/o Age w/Substances*) reflects the well‐documented age–prevalence relationship in adolescent drinking, where prevalence rises from ~10% at age 14 to over 70% by age 21 [1]. As Kinreich et al. [42] explicitly demonstrated, age is a critical confounding variable in ML models for AUD, noting that failure to properly control for age can lead to ‘misclassification of the model’ and biased classifications or predictions. Similarly, the inclusion of other substance use variables (tobacco, marijuana and other drugs) in models introduces potential information leakage, as these substances share common aetiological pathways with alcohol use and often co‐occur temporally [38, 43].

Our SHAP ranking of the Top 10 most important variables for our model revealed that the most informative variables for distinguishing adolescent drinkers from non‐drinkers fell into three clinically meaningful domains (Figure 8): alcohol expectancies, mental health symptoms and lifestyle characteristics. Notably, because our model was trained exclusively on clinical measures, with substance‐use variables removed and age‐related variance regressed out, the emergence of these interpretable, theory‐consistent predictors, rather than proxies for age or co‐occurring substance use, supports the construct validity of the model and suggests that it captures behavioural and psychosocial risk signals specific to alcohol involvement.

Alcohol expectancies, which refer to beliefs about the anticipated effects of drinking, emerged as particularly salient variables in our model, with expectancies regarding enhanced social behaviour, sexual enhancement and improved cognitive and motor abilities ranking among the top discriminating variables. This is consistent with previous research, demonstrating that positive alcohol expectancies were highly associated with drinking initiation, frequency and quantity during adolescence [44, 45], rather than merely reflecting current use, underscoring their value for early identification. The relationship is also reciprocal: As adolescents drink, reinforcing experiences strengthen these expectancies, producing a self‐perpetuating cognitive–behavioural cycle. That such expectancies were among the strongest predictors in a purely clinical model is clinically encouraging, as they are directly assessable in brief self‐report and are modifiable; expectancy‐challenge interventions that recalibrate positive beliefs have shown promise in reducing adolescent drinking [46, 47], making this domain an actionable screening and prevention target.

Mental health symptoms, including panic, OCD and PTSD, also emerged as important variables. Epidemiological research has established robust comorbidity between anxiety disorders and AUD, with anxiety disorders being two to three times more prevalent among individuals with alcohol problems than in the general population [48, 49]. This finding is also consistent with the self‐medication hypothesis, whereby adolescents may use alcohol to manage distressing symptoms [50], and with negative‐reinforcement (tension‐reduction) models in which the acute anxiolytic effects of alcohol reinforce continued use. Adolescence is a period of peak onset for anxiety and trauma‐related disorders, and the presence of panic and PTSD symptoms specifically may index a stress‐reactive pathway to drinking, in which alcohol serves as a maladaptive coping strategy for hyperarousal and intrusive symptoms [51, 52]. The contribution of OCD symptoms is less classically emphasized in the alcohol literature and may reflect a broader dimension of internalizing distress and compulsivity rather than a disorder‐specific effect; it warrants further study. Importantly, the association is likely bidirectional, as chronic alcohol exposure can exacerbate anxiety through neuroadaptation, potentially entrenching a symptom–drinking loop. Clinically, these findings argue for integrated, transdiagnostic screening in which adolescents presenting with anxiety or trauma symptoms are also evaluated for emerging alcohol risk, and vice versa.

Lifestyle characteristics reflecting adolescents' daily routines and social environments constituted the third major classification domain, encompassing sleep patterns, friend‐making, nighttime activities and spending money behaviours. Sleep disturbances have been increasingly recognized as both risk factors for and consequences of adolescent alcohol use [53]. The normative circadian delay of adolescence, combined with impaired emotion regulation and heightened reward sensitivity under sleep loss, may lower the threshold for impulsive drinking. Friend‐making is consistent with the extensive literature documenting peer friends' influence as one of the most proximal risk factors for adolescent substance use [54], operating through both peer selection and socialization, including modelling of drinking norms and increased access. Unstructured, unsupervised socializing with peers creates opportunities for risk‐taking behaviour, including substance use [55]. Finally, adolescent ways of spending money, relating to disposable income, have been identified as a significant predictor of drinking behaviour [56]. Adolescents with a higher level of money spending have greater access to alcohol and may participate in social contexts where drinking is more prevalent [57].

Several limitations should be acknowledged. First, although the sample size of 801 participants was adequate for the current analyses, it may limit the generalizability of our findings to more diverse adolescent populations. Second, formal external validation using an independent dataset was not conducted. Third, our age‐confound adjustment assumed linear age effects and did not account for potential non‐linear age–variable relationships. Future work will address these limitations by validating the proposed framework in larger and independent cohorts, adopting non‐linear or non‐parametric residualization (e.g., polynomial or spline terms, or generalized additive models) and assessing its effect on classification performance. In addition, we will extend our work to longitudinal analyses to prospectively predict drinking status across subsequent NCANDA follow‐up assessments.

## Author Contributions

**Ruobing Liu:** conceptualization, methodology, validation, formal analysis, data curation, writing – original draft, writing – review and editing, visualization. **Mohamed Azzam:** conceptualization, methodology, writing – review and editing, supervision, project administration. **Nicole L. Zabik:** conceptualization, writing – review and editing. **Shibiao Wan:** writing – review and editing, funding acquisition. **Jennifer Urbano Blackford:** conceptualization, writing – review and editing, funding acquisition. **Jieqiong Wang:** conceptualization, methodology, writing – review and editing, supervision, project administration, funding acquisition.

## Funding

Research reported in this publication was supported by the US National Science Foundation under Award Numbers 2500836 and 2614824 and the NIH Office of the Director under Award Numbers R03OD038391 and R03CA317707. This work was also partially supported by the National Institute of General Medical Sciences (NIGMS) under Award Numbers P20GM103427 and U54GM115458 and by the National Institute on Alcohol Abuse and Alcoholism (NIAAA) under Award Numbers R01 AA029127, P60 AA031124, R21AA032098, F32AA032170, L30AA032656, and P50AA030407‐5126 (Pilot Core grant). This study was in part financially supported by the Child Health Research Institute at UNMC/Children's Nebraska. This work was also partially supported by Nebraska EPSCoR FIRST Award (OIA‐2044049).

## Ethics Statement

All data used in this work originate from the National Consortium on Alcohol and Neurodevelopment in Adolescence (NCANDA), a multi‐site longitudinal study conducted at five institutions: the University of California San Diego, Duke University, the University of Pittsburgh, SRI International and Oregon Health & Science University. Each site received Institutional Review Board approval for the original protocol. Participants aged 18 or older provided written informed consent; minors provided assent in addition to parental consent. Because the present secondary analysis relies solely on de‐identified records, it qualifies as exempt from additional ethical review.

## Conflicts of Interest

The authors declare no conflicts of interest.

## Supporting information


**Table S1:** Shared protocol vs. model‐specific hyperparameters.

## Data Availability

Data for this study were obtained from the National Consortium on Alcohol and NeuroDevelopment in Adolescence (NCANDA), a publicly accessible dataset available through the NCANDA data sharing portal. The related codes and generated results are available from the corresponding author upon reasonable request.
