# Supplementary material for: Classification of Adolescent Drinking via Behavioral, Biological and Environmental Variables: A Machine Learning Approach With Bias Control
Source: Addict Biol. 2026 Sep 11;31(9):e70188. doi: 10.1111/adb.70188 (PMC13563688; doi:10.1111/adb.70188)
Supplement: Supplementary file 1 [file ADB-31-e70188-s001.docx]

**SUPPLEMENTARY MATERIAL**

**Table of contents**

**Supplementary methods**

- Methods S1. Participants
- Methods S2. Data dictionary
- Methods S3. Common experiment protocol

**References**

**SUPPLEMENTARY METHODS**

**Methods S1. Participants**

The definitions of non-drinkers and drinkers followed the modified Cahalan inventory ^1^ using responses from the Customary Drinking and Drug Use Record ^2^. Non-drinkers met the following criteria: 1) drinking frequency of less than once per month or less than once per year; 2) consumption of no more than three drinks per month; and 3) no binge drinking episodes. Drinkers encompassed both moderate and heavy drinking categories. Moderate drinkers were defined as those reporting: 1) low frequency with high quantity (e.g., less than once per month with three to four drinks on average and four to five drinks maximum); or 2) moderate frequency with low quantity (e.g., once per week with two drinks on average and less than four drinks maximum). Heavy drinkers were defined as those reporting: 1) moderate frequency with high quantity (e.g., twice per month with three to four drinks on average and more than four drinks maximum); or 2) high frequency with moderate consumption (e.g., once or more per week with one to four drinks on average and four or fewer drinks maximum). There were 9 participants without baseline data, and 21 participants without any follow-up data, excluded from analyses. Based on these definitions, the final 801 samples included 661 non-drinkers and 140 drinkers.

**Methods S2. Variable selection**

To ensure the reliability and independence of input variables for machine learning, we applied the following variable selection criteria: 1) removal of constant variables that showed no variability across participants; 2) exclusion of variables with >50% missing values; and 3) elimination of variables directly related to substance use, to prevent confounding or circularity in predicting drinking behavior. After applying these criteria, a total of 167 baseline variables across behavioral, biological, and environmental domains were retained for model development (Table 1). Alcohol expectancy (7 variables) assessed beliefs about alcohol effects using the Alcohol Expectancy Questionnaire (AEQ) ^3^. Family history (4 variables) captured familial substance use disorders via the Family History Assessment Module (FHAM) ^4^. Socioeconomic status (4 variables) included parental education, family income, and composite SES scores ^5^. Personality traits (5 variables) were measured using the Ten-Item Personality Inventory (TIPI) ^6^ and the UPPS-P Impulsive Behavior Scale (UPPS-P) ^7^. Environmental atmosphere (48 variables) encompassed neighborhood characteristics, parental monitoring, peer influences, and school climate ^8–10^. Child behavior and daily functioning (21 variables) assessed sleep patterns ^11–13^, executive function, and coping strategies using the Behavior Rating Inventory of Executive Function (BRIEF) ^14^ and Responses to Stress Questionnaire (RSQ) ^15^. Medical history (5 variables) included traumatic brain injury and hospitalizations ^16^. Demographics (5 variables) captured family structure and educational engagement ^17^. Physical measures (3 variables) included height, weight, and BMI ^17^. Pubertal development (2 variables) was assessed using the Pubertal Development Scale (PDS) ^18^. Peer relationships (1 variable) measured social support quality via the Social Support Questionnaire (SSQ) ^19^. Daily routine (31 variables) captured activities and competence ^17,20–22^. Psychiatric symptomatology (31 variables) assessed depression, conduct disorder, attention-deficit/hyperactivity disorder (ADHD), obsessive-compulsive disorder (OCD), posttraumatic stress disorder (PTSD), panic, and anxiety using the Semi-Structured Assessment for the Genetics of Alcoholism (SSAGA) ^23^ and Center for Epidemiologic Studies-Depression Scale (CES-D) ^24^.

**Methods S3. Common experiment protocol**

All six models were trained and evaluated under an identical, leakage-controlled protocol so that performance differences reflect the model and its training objective rather than differences in tuning effort. Each was applied to the same 25 data partitions (five repetitions of five-fold cross-validation; within every fold, the test and validation sets were class-balanced, and the training set retained the native class ratio). Features were median-imputed and, except for the random forest (which is scale-invariant), standardized with a StandardScaler; both transforms were fit on the training split only and applied to validation and test. Every model followed three phases: Phase A, hyperparameters, number of training epochs (where applicable), and the decision threshold were selected on the training to validation split, using validation AUC as the primary criterion (ties broken by validation F1) and the F1-maximizing threshold on the validation predicted probabilities; Phase B, the model was refit on the combined training+validation data with the Phase-A configuration frozen; Phase C, the refit model was evaluated exactly once on the untouched test set. All runs used a fixed random seed (42), and we report accuracy, F1, sensitivity, specificity, AUC, and log-loss. Model-specific hyperparameters and search spaces. FocalTab fine-tuned a TabPFN backbone (2 ensemble estimators, float32 inference, pre-training limits disabled) with focal loss, optimized by RAdam (meta-batch size 4, inference context up to 10,000 samples) for up to 80 epochs with the best epoch chosen on validation; the learning rate was selected on validation from {1×10⁻⁵, 5×10⁻⁶, 5×10⁻⁵, 1×10⁻⁴, 1×10⁻³, 5×10⁻³, 1×10⁻²}, and the focal-loss parameters were grid-searched over α ∈ {0.05, 0.10, …, 0.45} and γ ∈ {0.5, 1.0, …, 4.5} (81 combinations). The TabPFN baseline used the same backbone without gradient fine-tuning, selecting the number of ensemble estimators (8, 16) and softmax temperature (1.0, 0.7) on validation. The focal MLP and cross-entropy MLP were identical two-hidden-layer networks (128–64 units, ReLU, batch normalization, dropout 0.3, sigmoid output), trained with Adam (batch size 32) for up to 80 epochs with early stopping on validation AUC (patience 10, best weights restored) and the same learning-rate grid as FocalTab; the two differed only in the loss (focal, with the same α/γ grid, vs. binary cross-entropy). The random forest and logistic regression were tuned with 80-iteration randomized search scored by validation AUC: the forest over n_estimators ∈ {100–300}, max_depth ∈ {10, 15, 20, 25, 30, None}, min_samples_split ∈ {2, 5, 10, 15}, min_samples_leaf ∈ {1, 2, 4, 6}, max_features ∈ {√, log₂, 0.3, 0.5, 0.7}, max_samples ∈ {0.7, 0.8, 0.9, None}, and class_weight ∈ {balanced, balanced_subsample, None}; the logistic regression (saga solver, max_iter=5000) over C (30 values log-spaced in 10⁻⁴–10⁴), penalty ∈ {L1, L2, elastic-net}, l1_ratio ∈ {0.0, 0.1, …, 1.0}, and class_weight ∈ {balanced, None}. Class imbalance was addressed by each model's appropriate mechanism: focal loss for the focal models, class_weight for the random forest and logistic regression, and, additionally, the SMOTE and undersampling data variants evaluated in parallel.

Table S1. Shared protocol vs. model-specific hyperparameters

| Held identical (all models) | 25 splits (5×5 CV); median imputation + standardization (train-fit only); selection by validation AUC (F1 tie-break); F1-optimal threshold on validation; select, refit, single-test protocol; seed 42; same metrics. |
| --- | --- |
| FocalTab | TabPFN (2 est., float32); RAdam, meta-batch 4, ctx 10 000, ≤80 epochs; LR ∈ 7-value grid; α∈{0.05…0.45}, γ∈{0.5…4.5}. |
| TabPFN | TabPFN, no fine-tuning; n_estimators∈{8,16}, softmax_temperature∈{1.0,0.7}. |
| MLP (Focal) | 128–64, BN, dropout 0.3, Adam, batch 32, ≤80 epochs, early-stop on val AUC (patience 10); same LR grid; same α/γ grid. |
| MLP (BCE) | Identical to MLP-focal; loss = binary cross-entropy. |
| Random Forest | RandomizedSearch (80 iters, val AUC): n_estimators,max_depth,min_samples_{split,leaf},max_features,max_samples,class_weight. |
| Logistic Regression | RandomizedSearch (80 iters, val AUC): C, penalty {L1/L2/elastic-net}, l1_ratio, class_weight; saga, max_iter=5000. |

**REFERENCES**

1.	Cahalan, D., Cisin, I. H. & Crossley, H. M. *American Drinking Practices: A National Study of Drinking Behavior and Attitudes*. (Rutgers Center of Alcohol Studies, 1969).

2.	Brown, S. A. *et al.* Psychometric evaluation of the Customary Drinking and Drug Use Record (CDDR): a measure of adolescent alcohol and drug involvement. *Journal of studies on alcohol* **59**, 427–438 (1998).

3.	Brown, S. A., Christiansen, B. A. & Goldman, M. S. The Alcohol Expectancy Questionnaire: an instrument for the assessment of adolescent and adult alcohol expectancies. *Journal of studies on alcohol* **48**, 483–491 (1987).

4.	Rice, J. P. *et al.* Comparison of direct interview and family history diagnoses of alcohol dependence. *Alcoholism: Clinical and Experimental Research* **19**, 1018–1023 (1995).

5.	Hollingshead, A. B. Four Factor Index of Social Status. (1975).

6.	Gosling, S. D., Rentfrow, P. J. & Swann Jr, W. B. A very brief measure of the Big-Five personality domains. *Journal of Research in personality* **37**, 504–528 (2003).

7.	Cyders, M. A. *et al.* Integration of impulsivity and positive mood to predict risky behavior: development and validation of a measure of positive urgency. *Psychological assessment* **19**, 107 (2007).

8.	Tobler, A. L., Komro, K. A. & Maldonado-Molina, M. M. Relationship between neighborhood context, family management practices and alcohol use among urban, multi-ethnic, young adolescents. *Prevention Science* **10**, 313–324 (2009).

9.	Komro, K. A., Maldonado-Molina, M. M., Tobler, A. L., Bonds, J. R. & Muller, K. E. Effects of home access and availability of alcohol on young adolescents’ alcohol use. *Addiction* **102**, 1597–1608 (2007).

10.	California Department of Education. *California Healthy Kids Survey*. http://chks.wested.org (2012).

11.	Smith, C. S., Reilly, C. & Midkiff, K. Evaluation of three circadian rhythm questionnaires with suggestions for an improved measure of morningness. *Journal of Applied psychology* **74**, 728 (1989).

12.	Buysse, D. J., Reynolds III, C. F., Monk, T. H., Berman, S. R. & Kupfer, D. J. The Pittsburgh Sleep Quality Index: a new instrument for psychiatric practice and research. *Psychiatry research* **28**, 193–213 (1989).

13.	Wolfson, A. R. & Carskadon, M. A. Sleep schedules and daytime functioning in adolescents. *Child development* **69**, 875–887 (1998).

14.	Gioia, G. A., Isquith, P. K., Retzlaff, P. D. & Espy, K. A. Confirmatory factor analysis of the Behavior Rating Inventory of Executive Function (BRIEF) in a clinical sample. *Child neuropsychology* **8**, 249–257 (2002).

15.	Connor-Smith, J. K., Compas, B. E., Wadsworth, M. E., Thomsen, A. H. & Saltzman, H. Responses to stress in adolescence: measurement of coping and involuntary stress responses. *Journal of consulting and clinical psychology* **68**, 976 (2000).

16.	Corrigan, J. D. & Bogner, J. Initial reliability and validity of the Ohio State University TBI identification method. *The Journal of head trauma rehabilitation* **22**, 318–329 (2007).

17.	Brown, S. A. *et al.* The National Consortium on Alcohol and NeuroDevelopment in Adolescence (NCANDA): a multisite study of adolescent development and substance use. *Journal of studies on alcohol and drugs* **76**, 895–908 (2015).

18.	Petersen, A. C., Crockett, L., Richards, M. & Boxer, A. A self-report measure of pubertal status: Reliability, validity, and initial norms. *Journal of youth and adolescence* **17**, 117–133 (1988).

19.	Sarason, I. G., Sarason, B. R., Shearin, E. N. & Pierce, G. R. A brief measure of social support: Practical and theoretical implications. *Journal of social and personal relationships* **4**, 497–510 (1987).

20.	Achenbach, T. M. & Rescorla, L. A. *Manual for the ASEBA Adult Forms & Profiles*. (University of Vermont, Research Center for Children, Youth, & Families, Burlington, VT, 2003).

21.	Achenback, T. & Rescorla, L. A. Manual for the ASEBA school-age forms & profiles. *Burling: University of Vermont (Research center for children, youth and families)* (2001).

22.	Masten, A. S., Neemann, J. & Andenas, S. Life events and adjustment in adolescents: The significance of event independence, desirability, and chronicity. *Journal of Research on Adolescence* **4**, 71–97 (1994).

23.	Bucholz, K. K. *et al.* A new, semi-structured psychiatric interview for use in genetic linkage studies: a report on the reliability of the SSAGA. *Journal of studies on alcohol* **55**, 149–158 (1994).

24.	Radloff, L. S. The CES-D scale: A self-report depression scale for research in the general population. *Applied psychological measurement* **1**, 385–401 (1977).
